# G392E neuroserpin causing the dementia FENIB is secreted from cells but is not synaptotoxic

**DOI:** 10.1038/s41598-021-88090-1

**Published:** 2021-04-22

**Authors:** Thies Ingwersen, Christian Linnenberg, Emanuela D’Acunto, Shabnam Temori, Irene Paolucci, David Wasilewski, Behnam Mohammadi, Johannes Kirchmair, Robert C. Glen, Elena Miranda, Markus Glatzel, Giovanna Galliciotti

**Affiliations:** 1 Institute of Neuropathology, University Medical Center Hamburg-Eppendorf, Martinistrasse 52, 20246 Hamburg, Germany; 2 Department of Neurology, University Medical Center Hamburg-Eppendorf, Hamburg, Germany; 3 Department of Biology and Biotechnologies ‘Charles Darwin’, Sapienza University of Rome, Rome, Italy; 4 Centre for Molecular Informatics, Department of Chemistry, University of Cambridge, Cambridge, UK; 5 Department of Pharmaceutical Chemistry, Faculty of Life Sciences, University of Vienna, Vienna, Austria; 6 Division of Systems Medicine, Department of Metabolism Digestion and Reproduction, Imperial College London, London, UK; 7 Pasteur Institute – Cenci Bolognetti Foundation, Sapienza University of Rome, Rome, Italy

**Keywords:** Chemical biology, Neuroscience

## Abstract

Familial encephalopathy with neuroserpin inclusion bodies (FENIB) is a progressive neurodegenerative disease caused by point mutations in the gene for neuroserpin, a serine protease inhibitor of the nervous system. Different mutations are known that are responsible for mutant neuroserpin polymerization and accumulation as inclusion bodies in many cortical and subcortical neurons, thereby leading to cell death, dementia and epilepsy. Many efforts have been undertaken to elucidate the molecular pathways responsible for neuronal death. Most investigations have concentrated on analysis of intracellular mechanisms such as endoplasmic reticulum (ER) stress, ER-associated protein degradation (ERAD) and oxidative stress. We have generated a HEK-293 cell model of FENIB by overexpressing G392E-mutant neuroserpin and in this study we examine trafficking and toxicity of this polymerogenic variant. We observed that a small fraction of mutant neuroserpin is secreted via the ER-to-Golgi pathway, and that this release can be pharmacologically regulated. Overexpression of the mutant form of neuroserpin did not stimulate cell death in the HEK-293 cell model. Finally, when treating primary hippocampal neurons with G392E neuroserpin polymers, we did not detect cytotoxicity or synaptotoxicity. Altogether, we report here that a polymerogenic mutant form of neuroserpin is secreted from cells but is not toxic in the extracellular milieu.

## Introduction

Neuroserpin is a member of the serine protease inhibitor (serpin) superfamily secreted from neurons of the central and peripheral nervous system^1^. Previous research has established a role for neuroserpin in regulating proliferation and differentiation of neuronal precursor cells as well as maturation of synapses during hippocampal development^2^. In the adult brain, neuroserpin regulates synaptic plasticity and is neuroprotective during ischemic brain injury^3–5^. Neuroserpin is thought to exert its functions by inhibiting the proteolytic activity of tissue plasminogen activator (tPA)^6, 7^. As for most of the serpins, this occurs by the formation of a covalent complex after binding of tPA to the reactive center loop (RCL) of neuroserpin, resulting in the insertion of the cleaved RCL into β-sheet A of the serpin. This molecular mousetrap is a very efficient inhibitory mechanism, however the structural mobility that underlies it renders the serpin vulnerable to point mutations that alter its conformational stability, leading to the formation of an aberrant intermolecular link that drives the formation of polymeric chains of mutant serpin^8^. Six mutations have been identified so far in the neuroserpin gene that are responsible for amino acid exchanges in residues located in the region that regulates the opening of the β-sheet A, favoring polymer formation. These mutations cause a neurodegenerative disease called FENIB (familial encephalopathy with neuroserpin inclusion bodies), characterized by the accumulation of eosinophilic inclusion bodies composed of mutant neuroserpin polymers in neurons of different cortical and subcortical areas and in the spinal cord^9, 10^.

Although little is known about the pathological mechanisms leading to dementia in FENIB, studies over the past two decades have provided important information on the cellular response to mutant neuroserpin deposition. Cellular models of FENIB have been developed that recapitulate the polymerization and defective trafficking of mutant neuroserpin^11, 12^. FENIB mouse models expressing the Portland-S52R or Syracuse-S49P mutation have allowed to investigate the progressive accumulation of inclusion bodies and the resulting neuronal loss over time^13, 14^. Both cellular and mouse models provided evidence towards a fundamental concept of the serpinopathies asserting a correlation between tissue deposition of mutant neuroserpin and conformational instability triggered by each mutation. Moreover, they demonstrated that inclusion bodies accumulate within the ER and are predominantly composed of neuroserpin polymers. Whereas in mouse models transient unfolded protein response (UPR) and modest inflammation are triggered by mutant neuroserpin accumulation^14, 15^, in cellular models activation of NF-kappa B by the ER overload pathway in the absence of UPR has been reported^16, 17^. Importantly, in both model systems clearance of mutant neuroserpin via ER-associated protein degradation (ERAD) is crucial in limiting its accumulation and tissue deposition. In more recent years, investigations with a new cellular model system consisting of transgenic neural progenitor cells stably expressing neuroserpin carrying the highly polymerogenic G392E-mutation have evidenced a role for oxidative stress in the cellular toxicity underlying FENIB^18^. This study has revealed that genes protecting against oxidative stress are upregulated in the presence of mutant neuroserpin, and inhibition of the antioxidant response enhanced neuronal toxicity.

Using a HEK-293 cell model of FENIB overexpressing G392E-mutant neuroserpin, we disclosed how the lectin OS-9 delivers mutant neuroserpin to ERAD by binding to glycan side chains^19^. We noticed that, although mutant neuroserpin accumulated within the cells, there was a small fraction that was secreted into the culture medium. In the present study we assess how mutant neuroserpin is secreted, and whether this release into the culture medium exerts cell toxicity. We report that G392E-mutant neuroserpin, similarly to its wild-type counterpart, is secreted via the ER-to-Golgi pathway and we show that trafficking of the mutant, but not of the wild-type neuroserpin, can be pharmacologically regulated upon treatment with a small organic compound that potentially binds to β-sheet A of neuroserpin. By treating primary hippocampal neurons with recombinant G392E-mutant neuroserpin polymers or by co-culturing them with HEK-293 cells stably overexpressing the mutant serpin, we found no cytotoxicity or synaptotoxicity. Finally, we quantified an increased expression of the postsynaptic density protein 95 (PSD-95) in aged FENIB mice overexpressing Syracuse-S49P mutant neuroserpin.

In conclusion, we report that G392E-mutant neuroserpin is partially secreted from the cells, but the extracellular presence of the mutant serpin is not responsible for toxicity in our cell culture system.

## Materials and methods

### Animals

All animal procedures were performed in accordance with the ARRIVE guidelines, the EU Directive 2010/63/EU on the protection of animals used for scientific purposes and the institutional guidelines from the animal facility of the University Medical Center Hamburg Eppendorf. The animal procedures were approved by the local animal care committee (*Behörde für Lebensmittelsicherheit und Veterinärwesen*, ethical approval reference number ORG-739). Transgenic mice overexpressing either wild-type human neuroserpin (WT-Tg) or human neuroserpin carrying the S49P-Syracuse mutation (Syr-Tg) have been described before^13^, they were backcrossed to a C57BL/6J background for at least ten generations. Animals were kept in the animal facility of the University Medical Center Hamburg Eppendorf and maintained in groups of 2–4 littermates under standard housing conditions with food and water ad libitum. Mice of both sexes were used.

### Cell lines stably expressing neuroserpin

Human neuroserpin cDNA (wild-type and G392E-mutant) was cloned into the pcDNA3.1(-) vector (Thermo Fisher). HEK-293 cells (ATCC CRL-1573™) were maintained in DMEM High Glucose medium (Thermo Fisher) supplemented with 10% fetal bovine serum (PAA) at 37 °C and 5% CO_2_ and transfected with purified DNA (GeneJET PCR Purification Kit; Fermentas) using Lipofectamine 2000 (Thermo Fisher). Starting from 24 h after transfection, cells were grown in medium containing Geneticin (500 µg/ml, PAA) as selection agent. After 10–15 days, single clones were picked and expanded in separate wells. Stable overexpression of the neuroserpin protein was evaluated by western blot from 30–40 individual clones^19^. As a negative control, cells stably transfected with the empty vector were used.

### Primary hippocampal neurons

Hippocampi were dissected from C57BL/6J mice at birth in 10 mM glucose (Sigma) in phosphate buffered saline (PBS), tissues were minced and digested in glucose-PBS containing 0.5 mg/ml papain (Sigma) and 10 µg/ml DNase (Roche) for 30 min at 37 °C. The digested hippocampi were pelleted by centrifugation for 4 min at 1000×g, 37 °C and washed in Neurobasal medium (Thermo Fisher) supplemented with B-27 (Thermo Fisher), GlutaMAX (Thermo Fisher) and penicillin/streptomycin (Thermo Fisher) (Neurobasal+). The washing step was repeated for a total of four times. Tissues were triturated by pipetting 20 times up and down through a 1 ml tip, cells were plated on coverslips previously coated with 0.5 mg/ml poly-l-lysine (Sigma) at a concentration of 30,000–40,000 cells/cm^2^ in Neurobasal+ . In order to prevent the growth of glial cells, 10 µM of the mitotic inhibitor fluorodeoxyuridine (Sigma) was added to the medium one day after seeding the cells. Afterwards, half of the medium was changed every 4–5 days.

### Antibodies

The following antibodies were used for western blot: Anti-neuroserpin goat polyclonal antibody G64 (generation and affinity-purification have been previously described^13^) (0.5 ug/ml); Anti-synaptophysin (Abcam ab32594, 1:1000); Anti-SNAP25 (Abcam ab41455, 1:1000); Anti-neuroserpin (Abcam ab46761, 1:5000 and ab32901, 1:2000); Anti-synapsin-I (Cell Signaling Technology D12G5, 1:1000); Anti-PSD-95 (Millipore clone EP2652Y, 1:1000); Anti-beta-actin (Millipore clone C4, 1:5000); Anti-PDI (StressMarq SPC114C, 1:2000); Anti-GFP (Clontech mouse Living Colors monoclonal antibody 632,459, 1:5000). The following antibodies were used for immunofluorescence: Anti-MAP2 (Sigma M4403, 1:200); Anti-cleaved caspase 3 (R&D Systems AF835, 1:100); Anti-synaptophysin (Abcam ab32594, 1:200); Anti-PSD-95 (Millipore MAB1598, 1:100); Anti-neuroserpin goat polyclonal antibody G64 (3 ug/ml).

### Compound 1

Compound 1 (CAS 920626-34-2; 4-[[2-[(1-methyl-1H-tetrazol-5-yl)thio]acetyl]amino]-*N*-(4,5,6,7-tetrahydro-5-methylthiazolo[5,4-c]pyridin-2-yl)-benzamide; obtained from Enamine Ltd. – catalogue number Z30159011) was identified by docking the Enamine library of screening compounds against an X-ray structure of cleaved human neuroserpin (PDB 3F02) with residues E347 to V354 manually removed (these residues are part of strand 4 of β-sheet A). The Enamine screening library was obtained from the ZINC database^20^ and used as is for docking with GOLD version 5.0.1^21^. The binding site for docking was defined to include all residues within a 5 Å radius around the above mentioned removed residues. All settings and parameters for docking were kept default. GoldScore was employed for compound ranking.

### Cell treatments

HEK-293 cells were seeded at a concentration of 1.8 × 10^5^ cells per well in a 12-well plate and treated one day after plating. Brefeldin A (BFA) (Sigma) dissolved in ethanol was added at a concentration of 10 µg/ml in 600 ul Opti-MEM (Thermo Fisher) per well for 4 h at 37 °C, compound 1 was dissolved in DMSO and added at a concentration of 100 µM in 600 ul Opti-MEM per well for 18 h at 37 °C. For both BFA and compound 1 treatments, three independent experiments with three technical replicates each were performed (n = 3). Primary hippocampal neurons were treated at DIV14 by addition of 20 nM or 100 nM human recombinant neuroserpin (wild-type and G392E-mutant) and incubated for 18 h. In all experiments, negative control denotes treatment with solvent alone (PBS-10% glycerol). Alternatively, primary hippocampal neurons at DIV14 were co-cultured with HEK-293 cells stably expressing human neuroserpin (wild-type and G392E-mutant) for 18 h. HEK-293 cells were seeded one day before treatment (10^4^ cells/cm^2^) on coverslips (13-mm diameter) spotted with small wax dots. For treatment, the coverslips with HEK-293 cells were washed with PBS and placed in an inverted orientation on the coverslips with primary neurons in 500 ul Neurobasal+ medium. Presence of the wax dots allowed to maintain a distance between both coverslips. Four to six independent neuronal preparations were treated. Negative control denotes co-culture with HEK-293 cells stably transfected with empty vector.

For transient expression of USP19, HEK-293 cells at 80–90% confluency were transfected with a plasmid encoding for human mCitrine-USP19 (Addgene) using Lipofectamine 2000 (Thermo Fisher) and following the manufacturer’s instructions.

### Protein extraction

Cell culture media from HEK-293 cells were collected and cleared from insoluble material by centrifugation for 10 min at 16,000×g, 4 °C. Cells were washed with PBS and extracts were prepared by scraping and vortexing the cells in lysis buffer composed of 20 mM Tris–HCl, pH 7.5, containing 150 mM NaCl, protease inhibitor cocktail (complete, Mini, EDTA-free, Roche), PhosSTOP phosphatase inhibitor cocktail (Roche) and Triton X-100 to a final concentration of 1%. Extracts were cleared from insoluble material by centrifugation for 10 min at 16,000×g, 4 °C.

For protein extraction from mouse tissues, hippocampi from 64 week-old mice (4–5 animals per group) were homogenized in 200 µl of 20 mM Tris–HCl, pH 7.5, containing 150 mM NaCl, protease inhibitor cocktail (complete, Mini, EDTA-free, Roche) and PhosSTOP phosphatase inhibitor cocktail (Roche) using a dounce homogenizer as previously described^3^. Proteins were solubilized by addition of Triton X-100 to a final concentration of 1%. Extracts were cleared from insoluble material by centrifugation for 30 min at 20,000×g, 4 °C. Protein concentration was determined with Quick Start Bradford 1 × Dye Reagent (BioRad Laboratories) as described by the manufacturer. n = 4–5 and three technical replicates were performed.

### Western blotting and densitometry

HEK-293 cell culture media and cell extracts and 80 µg mouse hippocampal extracts were electrophoretically separated on 10% SDS-PAGE under reducing conditions. Alternatively, HEK-293 cell media and cell extracts were separated on 8% non-denaturing PAGE^19^. Proteins were transferred to nitrocellulose membranes (Bio-Rad Laboratories) and membranes were blocked for 1 h at RT with Roti-ImmunoBlock (Carl Roth) in Tris-buffered saline. Primary antibodies were incubated overnight at 4 °C in Tris-buffered saline containing 0.05% Tween-20 and Roti-ImmunoBlock. Secondary antibodies conjugated with IRDye 800CW or IRDye 680RD (LI-COR Biosciences, diluted 1:10,000) were incubated in the same buffer for 1 h at RT. Membranes were scanned using an Odyssey Infrared Imaging System (LI-COR Biosciences). Densitometric quantification was performed with LI-COR Odyssey Software, version 2.0 (LI-COR Biosciences) and local background subtraction. Band intensity was normalized to beta-actin or PDI expression. Alternatively, all proteins in the samples were quantified using Revert 700 Total Protein Stain (T.P.S) (LI-COR Biosciences).

### Non-denaturing PAGE and Coomassie staining of recombinant neuroserpin

Human recombinant wild-type and G392E-mutant neuroserpin were produced in *E. coli* with an N-terminal His-tag, affinity-purified and finally dissolved in PBS containing 10% glycerol (Biomatik). Both forms (1.5 µg) were electrophoretically separated on 8% non-denaturing PAGE as described above. The gels were then washed with distilled water for 5 min and stained with the GelCode Blue Stain Reagent (Thermo Fisher) for 60 min according to the manufacturer's protocol. Bands were visualized with ChemiDoc XRS and Quantity One software (BioRad).

### Immunocytochemistry

Primary hippocampal neurons were fixed for 10 min in 4% paraformaldehyde-4% sucrose in PBS. A wash step in PBS was followed by a blocking/permeabilization step in 1% bovine serum albumin in PBS supplemented with 0.25% Triton X-100. The primary antibody was diluted in blocking/permeabilization buffer and incubated overnight at 4 °C. Secondary antibodies conjugated to Alexa Fluor 555 and Alexa Fluor 488 (Thermo Fisher, dilution 1:500) were incubated for 1 h at RT. Coverslips were mounted on glass slides using Fluoromount-G with DAPI (SouthernBiotech), dried and pictures were taken using a Leica TCS SP5 confocal microscope, the Plan-APOCHROMAT 63 × oil-immersion lens and 1024 × 1024 pixels for frame. For quantification of caspase 3 cleavage, six independent neuronal preparations were treated with recombinant neuroserpin (20 nM), six pictures per coverslip were taken, and the number of nuclei positive to cleaved caspase 3 in relation to the total number of DAPI-positive nuclei was evaluated using the software ImageJ (NIH). For quantification of synapse toxicity, four to six independent neuronal preparations were treated with recombinant neuroserpin (20 nM and 100 nM) or co-cultured with HEK-293 cells. Pictures were taken from 10–12 neurons per coverslip. Density and area of synaptophysin-positive synaptic puncta along MAP2-positive dendrites was quantified using the software SynPAnal^22^.

### Immunohistochemistry

Mouse brains were post-fixed in 4% formaldehyde, processed for paraffin embedding and cut (4 µm) using standard protocols. Sections were deparaffinized, rehydrated and boiled for 10 min in 10 mM citrate buffer (10 mM sodium citrate, 0.05% Tween20, pH 6.0) for antigen retrieval, permeabilized 1 h in PBS-0.2% Triton X-100 and blocked for 1 h in PBS-0.4% Triton X-100–5% BSA. Sections were incubated for 18 h with primary antibody in blocking buffer containing 0.2% Triton X-100. Anti-rabbit, anti-mouse or anti-goat conjugated to Alexa Fluor 555, Alexa Fluor 488 and Alexa Fluor 647, respectively, were used as secondary antibodies. Sections were covered (Fluoromount-G with DAPI, SouthernBiotech), dried and pictures were taken using a Leica TCS SP5 confocal microscope, the Plan-APOCHROMAT 40 × oil-immersion lens and 1024 × 1024 pixels for frame.

### Cell toxicity assays

Release of lactate dehydrogenase into culture medium (LDH assay) was evaluated using LDH cytotoxicity detection kit (Takara) as described by the manufacturer. HEK-293 cells were seeded at a concentration of 0.3 × 10^5^ cells per well in a 96-well plate and tested 24 h after plating. LDH release from primary neurons was evaluated after 18 h treatment with recombinant neuroserpin. For both assays, 100 ul culture medium was used. Cell viability was assessed using CellTiter 96 Non-Radioactive Cell Proliferation Assay (MTT assay, Promega) and following the manufacturer’s instructions. HEK-293 cells were seeded at a concentration of 0.3 × 10^5^ cells per well in a 24-well plate and tested 48 h after plating. For investigation of HEK-293 cell toxicity, three experiments were performed (n = 3). For LDH assay with cultured neurons, six independent neuronal preparations were treated (n = 6).

### EndoH and PNGaseF treatments

HEK-293 cells were seeded at a concentration of 1.8 × 10^5^ cells per well in a 12-well plate in 800 ul medium. One day after plating, cell media were collected, cleared from insoluble material by centrifugation for 10 min at 16.000×g, 4 °C, and concentrated ten times using centrifugal filters (MWCO 10K) (VWR). Cell extracts were prepared as described above. Proteins were deglycosylated with either EndoH or PNGaseF (both from New England BioLabs) according to the manufacturer’s instructions. Finally, samples were analyzed by 10% SDS-PAGE under reducing conditions. The experiment was repeated three times.

### Data analysis

In all experiments, means + /− SD are reported. To evaluate the significance of differences between two experimental conditions (LDH and MTT assay with HEK-293 cells as well as for evaluation of western blot analysis in USP19 transfection) statistical comparisons among groups were determined using two sample t-test assuming unequal variance. To analyse the change in intracellular and extracellular neuroserpin levels after treatment with compound 1 and BFA, the mean ratio of the normalized density data (intracellular/extracellular) was compared. Hypothesis testing was conducted on logarithmized data using a two sample t-test assuming unequal variance (Welch’s t-test). To evaluate the significance of differences of three experimental conditions (quantification of caspase 3 activation, of synaptic toxicity, for LDH assay with primary neurons and for western blot analysis of mouse hippocampal extracts) one-way analysis of variance was applied. Post-hoc analysis was subsequentially performed to determine pairwise comparison in case of prior significance. For this, Tukey’s ‘Honest Significant Difference’ method was used correcting for multiple testing. n and p values are found in figure legends and in the Results section. Statistical significance was set at *p ≤ 0.05, **p ≤ 0.01 and ***p ≤ 0.001. The following statistical software were used: GraphPad Prism version 5.0 (GraphPad Software), Excel (Microsoft-Office), R statistical computing language (version 3.6.1).

## Results

### G392E-mutant neuroserpin is partially secreted from the cell

**Figure 1:**
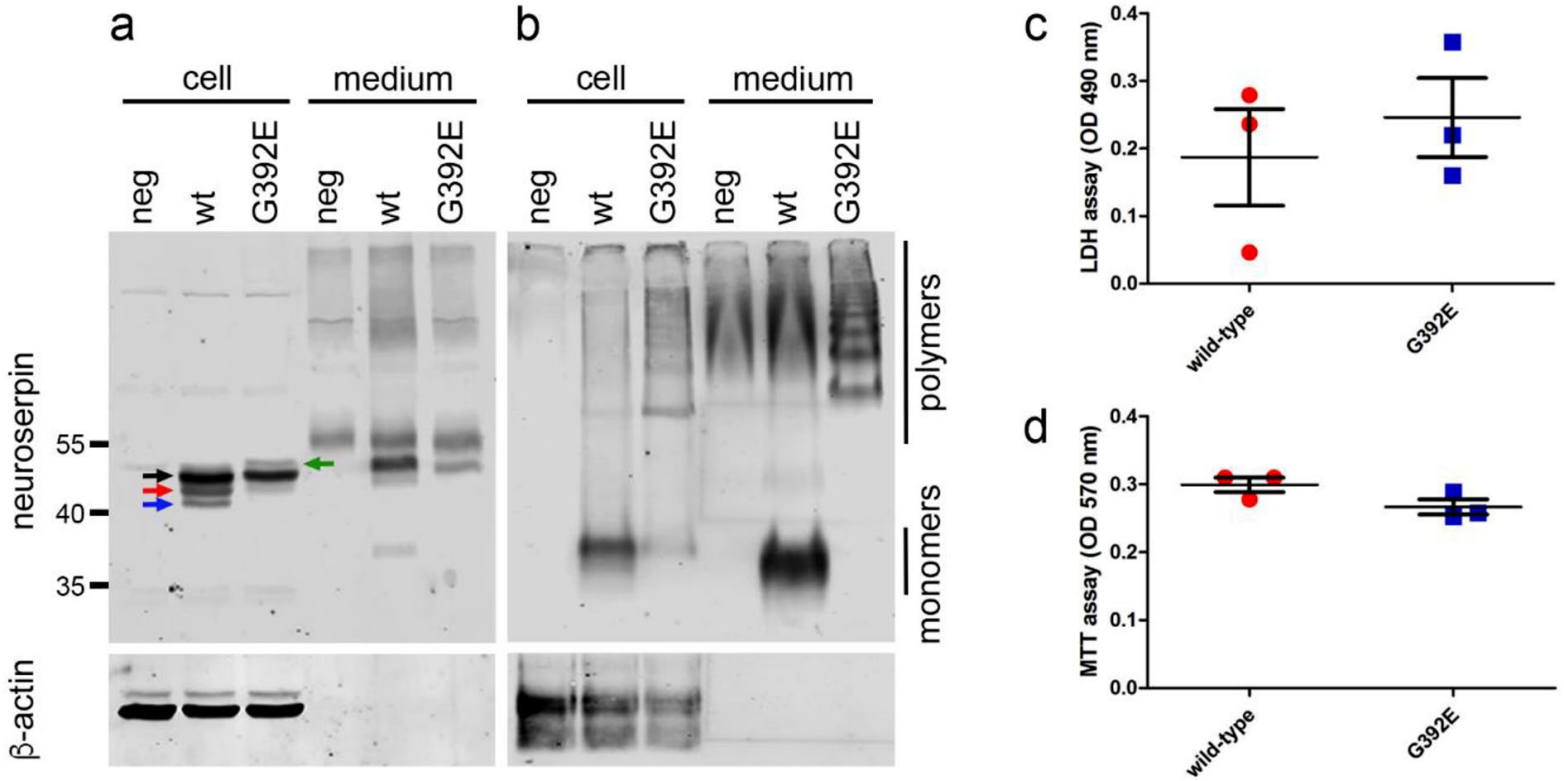
Overexpression of G392E-mutant neuroserpin in HEK-293 cells does not trigger cytotoxicity. HEK-293 cells were stably transfected with plasmids encoding human wild-type or G392E-mutant neuroserpin. Cell extracts (cell) and culture media (medium) were analyzed by SDS-PAGE (**a**) and non-denaturing PAGE (**b**) followed by western blot analysis with an antibody directed against neuroserpin. β-actin was used as loading control. The black arrow identifies the 50 kDa main form of neuroserpin, the red arrow a partially glycosylated form, the blue arrow the unglycosylated protein, and the green arrow G392E-mutant neuroserpin carrying an extra glycan chain on the asparagine residue 401. Cells stably transfected with empty vector were used as negative control (neg). Expression of the mutant form of neuroserpin did not lead to cell toxicity in HEK-293 cells, as shown in these cultures by quantification of LDH activity in the culture media (**c**, LDH assay) and cell proliferation (**d**, MTT assay). Western blot membranes shown in (**a**) and (**b**) have been cropped, full-length blots are presented in Supplementary Fig. S3. Values are mean + /− SD; n = 3; *p* = 0.5583 for LDH assay; *p* = 0.1029 for MTT assay.

In order to investigate the influence of mutant neuroserpin polymerization on the pathophysiology of FENIB, we used our previously established cell culture model for this disease^19^. HEK-293 cells were stably transfected with wild-type or G392E-mutant human neuroserpin. SDS-PAGE followed by western blot analysis revealed that wild-type neuroserpin is present in the cell as a main species of nearly 50 kDa (Fig. 1a, black arrow) plus two smaller species representing partially glycosylated (Fig. 1a, red arrow) and unglycosylated (Fig. 1a, blue arrow) neuroserpin. In contrast to the wild-type, for G392E-mutant neuroserpin the 50 kDa main form and a very faint band for the partially glycosylated form were detected. Moreover, a weak, higher band on top of the 50 kDa main form was observed (green arrow) that probably represents neuroserpin carrying a glycan structure on the asparagine residue 401, as described previously^23^. In the culture medium, a strong band for wild-type neuroserpin was observed, demonstrating the efficient secretion of the protein. Moreover, a faint band at approximately 38 kDa could also be identified. We also observed a weak band for the main species of G392E-mutant neuroserpin in the culture medium. Non-denaturing PAGE followed by western blot analysis demonstrated the presence of wild-type neuroserpin in its monomeric form in both the cell extract and the culture medium (Fig. 1b). G392E-mutant neuroserpin was found in both compartments as well, but in the cell extract only a faint monomeric band was detected, as most of the protein was found in the polymeric form. In the culture medium, monomers of mutant neuroserpin are absent, only slower migrating species forming a ladder of dimers, trimers, tetramers and bigger polymers were observed.

Although mutant variants of neuroserpin are known to polymerize and accumulate within the ER^13^, the presence of G392E-mutant neuroserpin in the culture medium has previously been described^12, 23^, but both the protein sorting pathway and potential harmful extracellular effects of mutant neuroserpin have never been reported. We first assessed if overexpression of G392E-mutant neuroserpin stimulates cell toxicity in our cellular model. For this purpose, release of lactate dehydrogenase into the culture medium (LDH assay) (Fig. 1c) and cell proliferation (MTT assay) (Fig. 1d) were compared between HEK-293 cells stably overexpressing wild-type and G392E-mutant neuroserpin. For both tests, we did not observe any significant difference between the two variants, concluding that presence of the mutant form of neuroserpin does not trigger major cytotoxicity in our cellular model (LDH assay: wild-type 0.187 + /− 0.12; G392E 0.246 + /− 0.10; *p* = 0.5583; MTT assay: wild-type 0.299 + /− 0.02; G392E 0.267 + /− 0.02; *p* = 0.1029).

### *G392E-mutant neuroserpin is released *via* the ER-to-Golgi secretory pathway*

**Figure 2:**
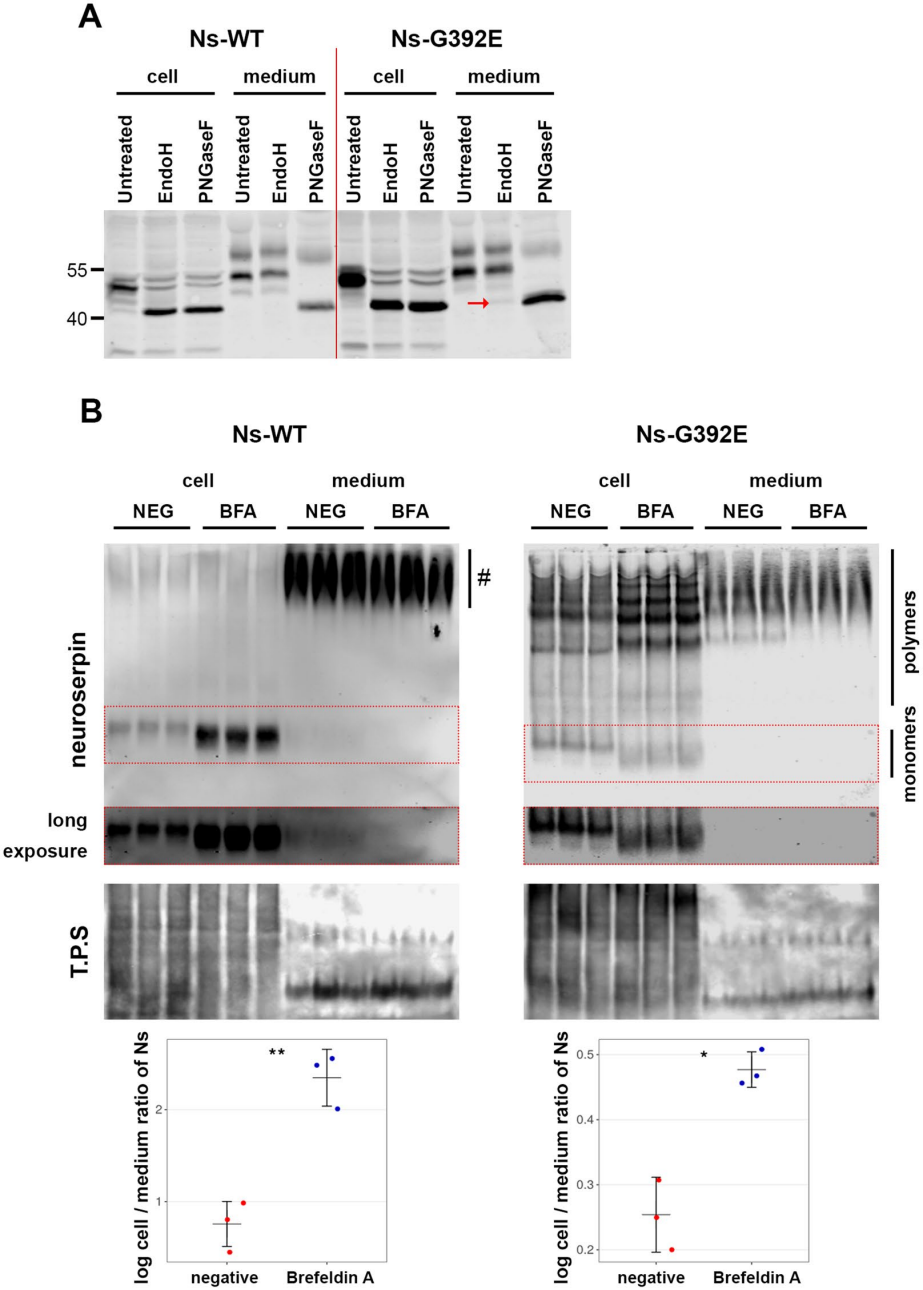
G392E-mutant neuroserpin is secreted via the ER-to-Golgi secretory pathway. (**A**) Cell extracts (cell) and culture media (medium) from HEK-293 cells overexpressing wild-type or G392E-mutant neuroserpin were collected and treated with EndoH or PNGaseF to remove N-linked mannose-rich or all types of N-linked oligosaccharides, respectively. In the cell extracts, both wild-type and mutant neuroserpin were completely deglycosylated by treatment with both enzymes. In the media, however, whereas wild-type neuroserpin acquired EndoH resistance, a faint band of Endo H-sensitive mutant neuroserpin could be detected (red arrow). The result shown is representative of three independent experiments. (**B**) HEK-293 cells stably transfected with wild-type or G392E-mutant neuroserpin were treated with BFA or solvent alone (NEG) for 4 h. Cell extracts (cell) and culture media (medium) were collected and analyzed by non-denaturing PAGE and western blot using an antibody directed against neuroserpin. As loading control, all proteins were stained (total protein stain, T.P.S). For wild-type neuroserpin, strong intracellular accumulation was observed upon treatment. Since culture medium was collected after a short incubation of 4 h, reduced secretion of monomeric neuroserpin was visible only after long exposure of the membrane (middle panel). Smears visible in the medium of wild-type neuroserpin-transfected cells (#) represent background signal, as this was also observed in cells transfected with vector only (see Fig. 1b). Similarly, mutant neuroserpin was increasingly retained within the cells treated with BFA, leading to enhanced polymerization, and less polymeric forms were detected in the media. For quantification, the mean ratio of the normalized density data (cell/medium) was compared. n = 3; *p* = 0.0027 for wild-type; *p* = 0.0104 for G392E-mutant. Three independent experiments with three technical replicates each were performed and a representative one is shown. Western blot membranes shown in (**a**) and (**b**) have been cropped, full-length blots are presented in Supplementary Fig. S3.

We then investigated whether mutant neuroserpin, similarly to its wild-type counterpart, is transported along the ER-to-Golgi secretory pathway. First, we collected culture media and cell extracts from HEK-293 cells stably transfected with wild-type and G392E-mutant neuroserpin and deglycosylated the proteins either with *N*-glycosidase F (PNGaseF), removing all types of N-linked oligosaccharides, or with endoglycosidase H (EndoH), removing N-linked mannose-rich oligosaccharides (Fig. 2A). Western blot analysis of the treated cell extracts revealed a shift in the mobility of both wild-type and G392E-mutant neuroserpin upon PNGaseF and EndoH treatments, demonstrating the presence of high-mannose type oligosaccharides on neuroserpin, irrespective of the G392E mutation. In the culture medium, secreted wild-type neuroserpin was EndoH-insensitive, thus demonstrating its passage through the Golgi. The band pattern of mutant neuroserpin was very similar to the wild-type protein, with the exception of a very faint band of EndoH-deglycosylated protein (Fig. 2A, red arrow). We postulated that this band could represent mutant neuroserpin secreted by the misfolded-associated protein secretion (MAPS) unconventional pathway^24^. In order to test this hypothesis, we transiently overexpressed USP19 (ubiquitin specific peptidase 19, a protein that promotes this secretory pathway) in HEK-293 cells stably transfected with G392E-mutant neuroserpin and quantified neuroserpin secretion (Supplementary Fig. S1). Overexpression of USP19 had no effect on mutant neuroserpin secretion (negative control: 41.63 + /− 21.53% intracellular; 58.37 + /− 21.53% culture medium; USP19: 45.63 + /− 17.05% intracellular; 54.37 + /− 17.05% culture medium; *p* = 0.6675). Therefore, our results suggest that the vast majority of G392E-mutant neuroserpin is secreted through the Golgi apparatus, although a small part of it may leave the cells via unconventional secretion different to MAPS, which is not involved in mutant neuroserpin release.

In order to corroborate our findings, we treated the stably transfected HEK-293 cells with brefeldin A (BFA), a drug that inhibits protein trafficking between the ER and the Golgi apparatus. Western blot analysis of cells transfected with wild-type neuroserpin showed accumulation of the neuroserpin monomer within the cells, and simultaneously reduced secretion into the culture medium (negative control: 0.76 + /− 0.24; BFA: 2.35 + /− 0.31 (log intracellular/extracellular ratio); *p* = 0.0027) (Fig. 2B, left panels and graph). Similar results were obtained upon BFA-treatment of HEK-293 cells overexpressing G392E-neuroserpin: an increase in the intensity of intracellular neuroserpin and a concomitant decrease in mutant neuroserpin secretion (negative control: 0.25 + /− 0.06; BFA: 0.48 + /− 0.03; *p* = 0.0104) (Fig. 2B, right panels and graph). Interestingly, not only the distribution of mutant neuroserpin was affected by BFA-treatment, but also its polymerization, as we observed a reduction in the monomeric form and an intensification of neuroserpin polymers in the intracellular fraction of cells treated with BFA. These results further support that G392E-mutant neuroserpin is secreted via the ER-to-Golgi pathway.

### Secretion of G392E-mutant neuroserpin can be pharmacologically regulated

**Figure 3:**
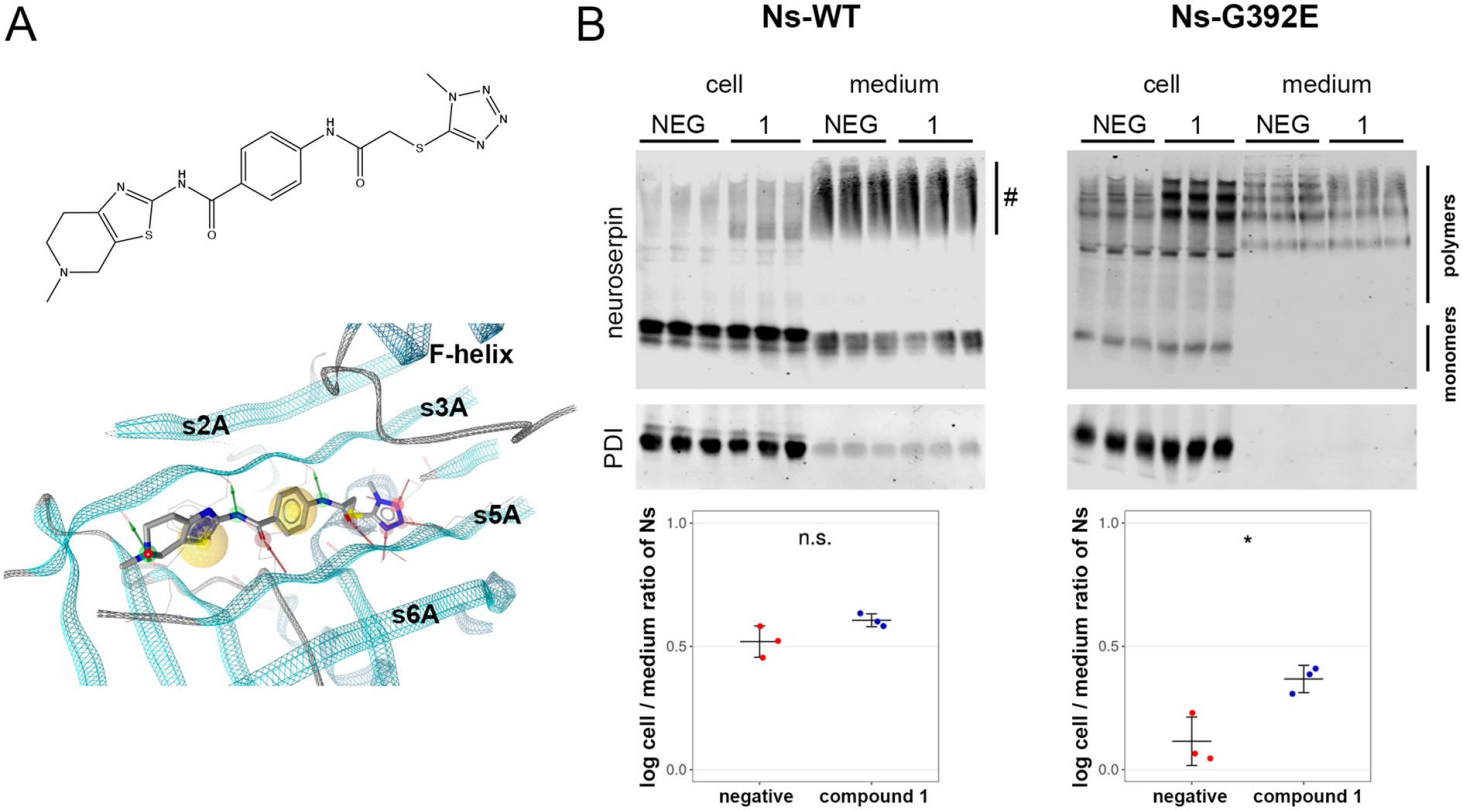
Secretion of G392E-neuroserpin can be pharmacologically regulated. (**A**) 2D structure (top) of compound 1 and visualization of its predicted binding mode (bottom). The protein backbone of neuroserpin is visualized as a ribbon. Green and red arrows indicate hydrogen bonds predicted to be formed between the ligand and the protein, and yellow spheres mark areas in the compound predicted to engage in hydrophobic interactions. Visualization with LigandScout^37^. (**B**) HEK-293 cells stably transfected with wild-type or G392E-neuroserpin were treated with the small organic compound 1 or solvent alone (NEG) for 18 h. Cell extracts (cell) and culture media (medium) were collected and analyzed by non-denaturing PAGE followed by western blot using an anti-neuroserpin antibody. PDI was used as loading control. Smears visible in the medium of wild-type neuroserpin-transfected cells (#) represent background signal, as they were also observed in cells transfected with vector only (see Fig. 1b). For quantification, the mean ratio of the normalized density data (cell/medium) was compared. n = 3; *p* = 0.1292 for wild-type; *p* = 0.0277 for G392E-mutant. Two independent experiments with three technical replicates each were performed and a representative one is show. Western blot membranes shown in (**B**) have been cropped, full-length blots are presented in Supplementary Fig. S3.

Compound 1 (Fig. 3A) is a small organic compound that was identified as a result of a structure-based virtual screening campaign for potential competitive inhibitors of the RCL insertion of neuroserpin. Since RCL insertion into β-sheet A is central not only for the formation of a stable complex with the target protease but also for the process of polymer formation, we assessed if an organic compound potentially blocking RCL insertion influences neuroserpin secretion^8^. Western blot analysis showed that the incubation of HEK-293 cells stably overexpressing wild-type neuroserpin with compound 1 did not influence the intra/extracellular distribution of the protein (negative control: 0.52 + /− 0.06; compound 1: 0.61 + /− 0.03 (log intracellular/extracellular ratio); *p* = 0.1292) (Fig. 3B). However, treatment of HEK-293 stably transfected with G392E-mutant neuroserpin led to an increase in intracellular retention of the protein, especially in its polymeric form, and a concomitant reduction of its presence in the culture medium (negative control: 0.12 + /− 0.10; compound 1: 0.37 + /− 0.06; *p* = 0.0277). These results indicate that compound 1 is a useful tool to specifically regulate the secretion of this mutant form of neuroserpin.

### Extracellular G392E-mutant neuroserpin polymers do not cause toxicity to primary hippocampal neurons

**Figure 4:**
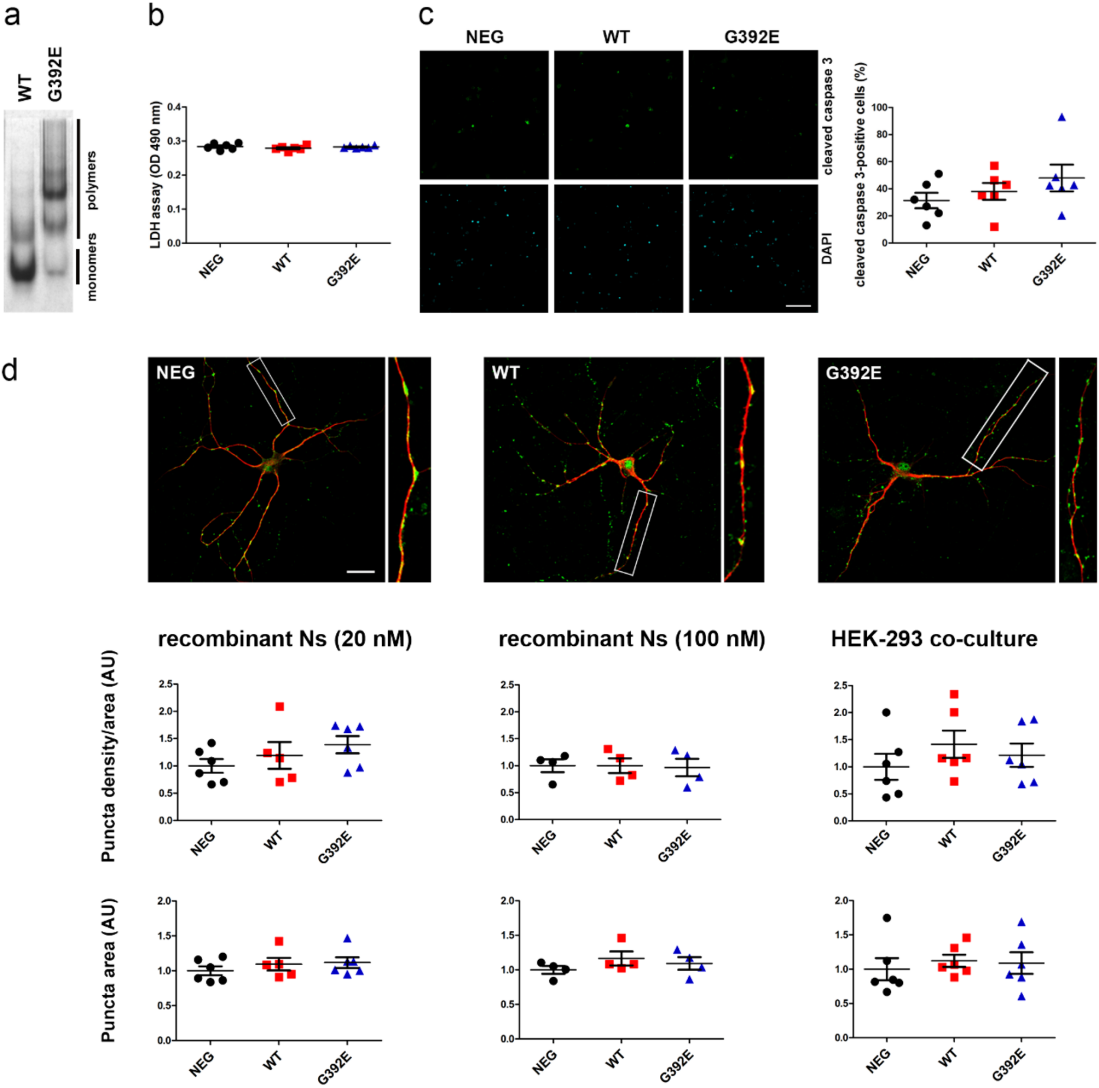
Extracellular G392E-mutant neuroserpin polymers are not toxic on cultured neurons. Primary hippocampal neurons at DIV14 were treated for 18 h with human recombinant neuroserpin (wild-type or G392E-mutant) or solvent alone (NEG). Six independent neuronal preparations were treated (n = 6). (**a**) Non-denaturing PAGE followed by Coomassie staining of human recombinant wild-type and G392E-mutant neuroserpin. (**b**) Culture media were collected and analyzed for LDH activity (LDH assay). No differences between groups were detected (*p* = 0.4472). (**c**) Treated neurons were stained with an antibody against cleaved caspase 3. Pictures were taken from six fields for each condition. Quantification of positive cells as percentage of total DAPI-positive cells did not reveal increase in apoptosis upon treatment (*p* = 0.3146). (**d**) Representative immunocytochemical staining of treated neurons to reveal MAP2-positive dendrites (red fluorescence) and synaptophysin-positive synaptic puncta (green fluorescence). Neurons were treated either with human recombinant wild-type or G392E-mutant neuroserpin (20 or 100 nM) or by co-culturing them with HEK-293 cells overexpressing either wild-type or G392E-mutant neuroserpin. Pictures were taken from 10–12 neurons for each condition. Density of synaptic puncta as well as synaptic puncta area were quantified using the software SynPAnal, value for the negative control was set to 1 (AU, arbitrary units). No significant differences between groups were found (for p-values, s. Supplementary Table S1). Scale bars: 75 µm in (**c**), 25 µm in (**d**).

Polymerogenic mutant neuroserpin is toxic to neuronal cells in cell culture models^18^ and in vivo^13^, however the mechanism leading to neuronal cell death is still obscure. We hypothesized that accumulation of extracellular mutant neuroserpin polymers could play a role in cell toxicity and tested this on cultured hippocampal neurons. Primary neurons were prepared from mouse hippocampi and allowed to develop dendrites and synaptic contacts for two weeks. At DIV14, neurons were treated by addition of 20 nM recombinant human G392E-mutant neuroserpin. As a control, we incubated cells with the same amount of recombinant human wild-type neuroserpin and with solvent alone. As shown in Fig. 4a, recombinant wild-type neuroserpin is mainly present in its monomeric form, whereas the G392E-variant mostly consists of polymers. After an 18 h incubation, neuronal cell toxicity was measured with three different methods. First, using the LDH assay we detected levels of LDH activity in the culture media of cells treated with both forms of neuroserpin very similar to those obtained from cells treated with solvent alone, thus excluding a detectable increase in cell toxicity due to the treatment with mutant neuroserpin (Fig. 4b and Supplementary Table S1). Second, treated neurons were stained with an antibody directed against active caspase 3, a protease that is activated by cleavage during cell apoptosis. The percentage of cleaved caspase 3-positive neurons in relation to the total amount of cells was quantified and we did not observe a significant increase in apoptosis between treatments (Fig. 4c and Supplementary Table S1). Third, we investigated whether the presence of extracellular mutant neuroserpin led to an increase in synaptotoxicity. In order to visualize dendrites and synaptic puncta, we stained treated neurons with antibodies directed against MAP2 and synaptophysin, respectively, and quantified the area of synaptophysin-positive synaptic puncta along MAP2-positive dendrites (Fig. 4d and Supplementary Table S1). We did not observe any significant difference in density or area occupied by synaptophysin-positive puncta between cells treated with wild-type or mutant neuroserpin or with solvent alone. We increased the concentration of recombinant human neuroserpin used for the treatment up to 100 nM, but this did not lead to detectable synaptotoxicity on primary hippocampal neurons (Fig. 4d and Supplementary Table S1). Finally, due to the importance of glycosylation in neuroserpin polymerization in FENIB^23, 25^, we exposed neuronal cultures to an alternative source of fully glycosylated neuroserpin by co-culturing the primary neurons with HEK-293 cells stably overexpressing and secreting either wild-type or G392E-mutant neuroserpin for 18 h. Again, no significant differences in density or area of synaptic puncta were observed (Fig. 4d and Supplementary Table S1). Taken together, these data support that, although present in the extracellular milieu, secreted mutant neuroserpin polymers are neither toxic to neurons nor lead to subtle synaptic degeneration under the conditions tested.

### Increased expression of the postsynaptic marker PSD-95 in the hippocampus of a FENIB mouse model

**Figure 5:**
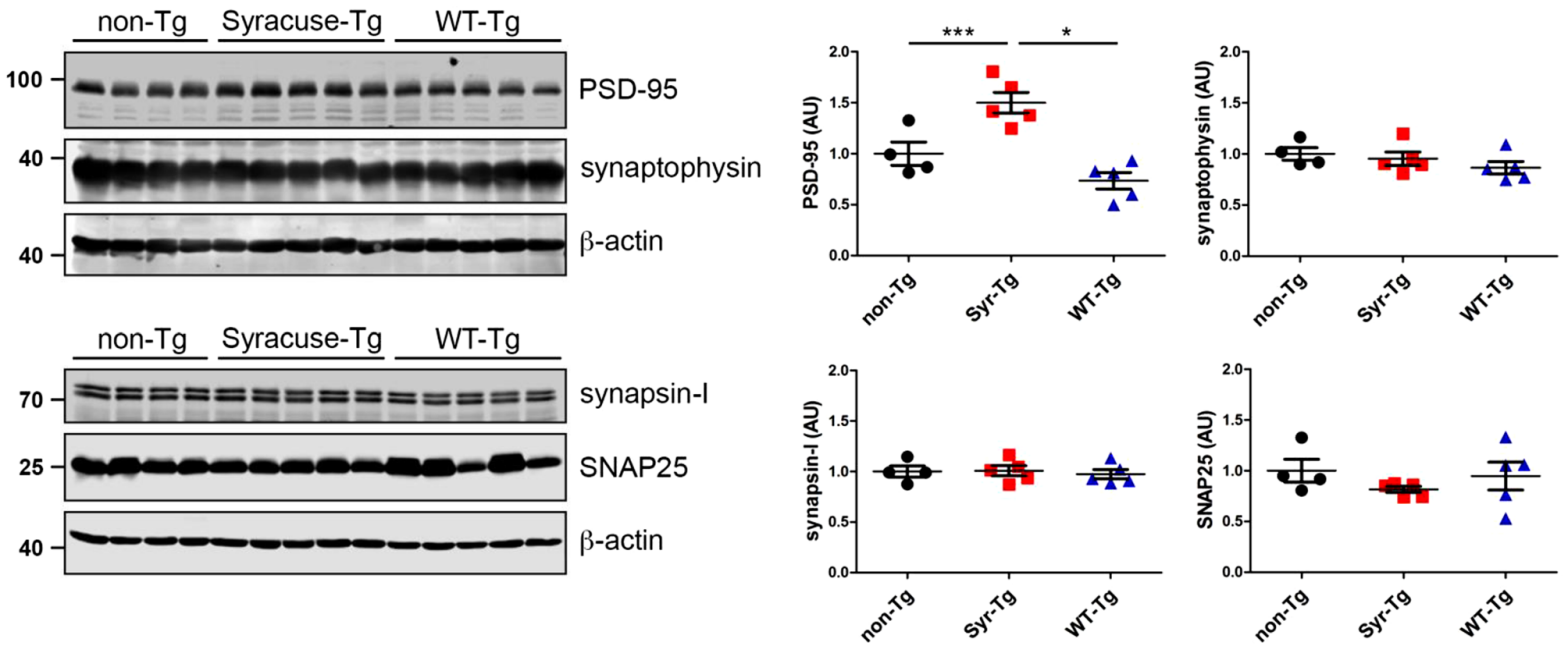
Increased expression of the postsynaptic marker PSD-95 in the hippocampus of mice overexpressing the S49P-Syracuse mutation. Representative western blots of hippocampal extracts from four-five different animals aged 64 weeks overexpressing either wild-type (WT-Tg) or S49P-Syracuse mutant neuroserpin (Syr-Tg). Non-transgenic, wild-type mice (non-Tg) were used as controls. Extracts were separated by SDS-PAGE and analyzed with antibodies against PSD-95 (post-synapse) and synaptophysin, SNAP25 and synapsin-I (pre-synapse). Band intensity was normalized to β-actin expression. Relative expression is presented (AU, arbitrary units), wild-type was set to 1 (mean + /− SD; n = 4–5; for PSD-95: non-Tg vs. Syr-Tg: *p* = 0.0003; non-Tg vs. WT-Tg: *p* = 0.1890; WT-Tg vs. Syr-Tg: *p* = 0.0118; *p* = 0.3547 for synaptophysin; *p* = 0.8795 for synapsin-I; *p* = 0.4594 for SNAP25). Western blot membranes have been cropped, full-length blots are presented in Supplementary Fig. S3.

Generation and characterization of a FENIB mouse model has previously been described^13, 14^. S49P-Syracuse mutant neuroserpin is overexpressed in the brain, neuroserpin polymerization, intracellular accumulation, and neuronal loss are the consequences. Here, we evaluated if synaptotoxicity can be detected as a result of mutant-neuroserpin expression. Immunohistochemical stainings evidenced the presence of neuroserpin-positive inclusion bodies in the CA1 region of the hippocampus of S49P-Syracuse transgenic mice at 45 and 80 weeks of life but not in mice overexpressing wild-type neuroserpin, whereas very similar staining pattern for pre-synaptic marker synaptophysin and post-synaptic marker PSD-95 were observed (Supplementary Fig. S2). In order to assess more subtle differences in synaptic composition, we analyzed by western blot the levels of four synaptic markers, PSD-95 (post-synapse), synaptophysin, synapsin-I and SNAP25 (pre-synapse) in the hippocampi of mice at 64 weeks of age, a time point at which massive accumulation of neuroserpin polymers and neuronal loss can be detected in this brain region^14^ (Fig. 5). As a control, we analyzed the hippocampus of transgenic mice overexpressing wild-type human neuroserpin, as well as non-transgenic control mice. Densitometric quantification revealed an increase in expression of PSD-95 (non-Tg: 1.00 + /− 0.23; Syr-Tg: 1.50 + /− 0.22; WT-Tg: 0.73 + /− 0.18; non-Tg vs. Syr-Tg: *p* = 0.0003; non-Tg vs. WT-Tg: *p* = 0.1890; WT-Tg vs. Syr-Tg: *p* = 0.0118). In contrast, levels of synaptophysin (non-Tg: 1.00 + /− 0.12; Syr-Tg: 0.95 + /− 0.15; WT-Tg: 0.87 + /− 0.14; *p* = 0.3547), synapsin-I (non-Tg: 1.00 + /− 0.11; Syr-Tg: 1.01 + /− 0.11; WT-Tg: 0.97 + /− 0.10; *p* = 0.8795) and SNAP25 (non-Tg: 1.00 + /− 0.23; Syr-Tg: 0.82 + /− 0.07; WT-Tg: 0.95 + /− 0.31; *p* = 0.4594) were unchanged.

## Discussion

Although many efforts have been made to elucidate the pathological mechanisms underlying neurodegeneration in FENIB, only a few aspects of the disease have been revealed. In particular, research has focused on pathways controlling the fate of the mutant protein after being retained within the ER. Using a cell model of FENIB consisting in HEK-293 cells overexpressing wild-type or G392E-mutant neuroserpin, we characterize here the secretion of this highly polymerogenic variant. The secretion of mutant serpin polymers causing other serpinopathies has been reported previously: extracellular polymers of α1-antitrypsin carrying the Z mutation (Z α1-AT, Glu342Lys) are present in the circulation of patients suffering from α1-AT deficiency^26^, and, similarly to what we show here for mutant neuroserpin, they have been shown to be secreted via the ER-to-Golgi pathway in cell culture studies^27^. Our deglycosylation studies, which demonstrate a similar maturation of neuroserpin glycans for both released variants investigated here, suggest secretion of G392E-neuroserpin through the Golgi apparatus. The very low amount of EndoH-sensitive G392E-mutant neuroserpin found in the culture medium could suggest that a small part of G392E-neuroserpin exits the cell via a Golgi-independent unconventional secretion pathway like MAPS^24^. This pathway has been described for cytoplasmic proteins, but since mutant neuroserpin is retrotranslocated to the cytosol for proteasomal degradation, we hypothesized that a small part of it could be released from the cells through this route. In our hands, overexpression of USP19, a deubiquitinase with chaperone activity that promotes MAPS, did not cause an increase in release of mutant neuroserpin, suggesting that MAPS is not involved in G392E-mutant neuroserpin secretion in our cell model. Still, we cannot exclude that other unconventional pathways are involved in secretion of mutant neuroserpin. Alternatively, although our data did not reveal an increase in cytotoxicity upon overexpression of the G392E-mutant neuroserpin in HEK-293 cells, we cannot completely exclude that cell death, at basal level and under the detection limit, may be responsible for the release in small amount of the EndoH-sensitive mutant neuroserpin in the culture medium.

We also found that secretion of G392E-mutant neuroserpin was reduced when cells were treated with BFA, further supporting the trafficking of G392E-mutant neuroserpin through the Golgi apparatus, and after treatment with the small chemical compound 1, a molecule selected in silico as a potential competitive inhibitor of the RCL insertion of neuroserpin. Our work here does not address the mechanism of compound 1, but we speculate that this molecule, although first selected to reduce mutant neuroserpin polymerization, rather facilitates polymerization of the mutant variant and thus reduces its secretion. Alternatively, enhanced polymerization could be a consequence of increased intracellular concentration of mutant neuroserpin within the ER due to inhibition of secretion, as observed upon BFA treatment, where the retained protein is mostly present as polymers, and as seen before in vitro^28^. With the BFA treatment, a shift of the monomeric band for G392E-mutant neuroserpin was also observed, compatible with the transition of a small fraction of mutant monomer to the latent state^29^.

A common feature of neurodegenerative diseases is the aggregation and tissue deposition of aggregation-prone proteins or fragments derived from them, e.g. Aβ in Alzheimer’s disease, α-synuclein in Parkinson’s disease, and PrP^Sc^ in spongiform encephalopathies^30^. FENIB is a neurodegenerative dementia caused by polymerization and tissue accumulation of neuroserpin protein whose conformation has been destabilized by a point mutation. Another shared characteristic of many dementias is synaptic loss at early stages of the disease, before neuronal loss is detected, which presents the highest correlation with cognitive impairment. The aggregated proteins themselves, mainly in the form of neurotoxic oligomers, can be responsible for the synaptic pathology, as in the case of Aβ and tau that deposit together at post-synaptic terminals in mice^31^. Neuroserpin is produced by neurons, gets transported along dendrites and axons and accumulates at the synapse^32^. In fact, neuroserpin-deficient mice have a synaptic phenotype with alterations starting during brain development and enduring in the adulthood, emphasizing the important function of neuroserpin at the synapse^2, 3^. Since our results showed that mutant neuroserpin is secreted through the same pathway as its wild-type counterpart, we hypothesized that it may be present at the synapse and act synaptotoxic. If this was true, then in FENIB, as in other dementias, neurodegeneration could start at the synapse. For this reason, we explored the potential toxicity of extracellular mutant neuroserpin to cells and synapses.

The extracellular toxicity of a polymeric serpin has already been reported for α1-AT deficiency, a pathology where polymers are thought to contribute to lung inflammation as a result of their neutrophil chemoattractant activity^33^. In contrast, we did not find cell toxicity by extracellular G392E-mutant neuroserpin polymers in our assays. The lack of cell toxicity on HEK-293 cells is not unexpected, since previous cell models of FENIB have shown that cells tolerate well the overexpression of mutant neuroserpin and the presence of polymers in the culture medium^11, 12^. To address toxicity in post-mitotic neurons, we used primary, fully differentiated hippocampal neurons and treated them with recombinant G392E-mutant neuroserpin used at 20 nM, a concentration of the serpin shown before to be, in its wild-type form, well tolerated by primary hippocampal neurons and protective against oxidative stress^34^, or 100 nM. To address the possibility that glycosylation plays a role in the extracellular effects of neuroserpin, maybe through an increase in polymer formation as reported before^23, 25^, we extended our analysis by co-culturing primary neurons with HEK-293 cells stably expressing wild-type or G392E-mutant neuroserpin. We used a density of HEK-293 cells that was well tolerated by neurons and similar to experiments where extracellular polymers were easily detectable. None of these conditions led to an increase in LDH release or to caspase 3 activation, demonstrating the absence of neuronal death. These results support the notion that neuronal death observed in FENIB is rather caused by intracellular stress and toxicity pathways activated by the accumulation of neuroserpin polymers within the ER, as reported in a neuronal model in vitro where overexpression of G392E-mutant neuroserpin led to an increase in oxidative stress^18^. Previous work from our group and others has reported synaptotoxicity on cultured neurons upon treatment with Aβ or prion protein^35, 36^, so we evaluated the effects of the treatments described above on synaptic phenotype. Despite using an approach that has proved successful for other protein aggregates, we did not observe any changes in any synaptic parameter tested. There may be several reasons for our results: mutant neuroserpin may be non-synaptotoxic and the neurodegenerative phenotype observed in FENIB is rather the result of the intracellular accumulation of aberrant polymers; or there are limitations in our experimental conditions, particularly with regards to the duration of the treatments. In both settings, we treated the cultures for 18 h because neurons did not tolerate a longer incubation, neuronal death was observed irrespectively of the treatment (wild-type or G392E-mutant neuroserpin) upon longer incubation. However, since dementias are disorders that develop over a long time, it is possible that short incubations do not trigger the toxic effects of extracellular polymers. Also, since we tested toxicity on pure neuronal preparations, we cannot exclude that the full complexity of the brain, with the presence of different cell types and factors produced by them, is needed to elicit harmful effects on synapses.

In order to portray a possible synaptic phenotype in vivo, we quantified synaptic markers in mice overexpressing S49P-Syracuse mutant neuroserpin at one year of age, a period when neuroserpin-positive inclusion bodies accumulate exponentially^13, 14^. Whereas pre-synaptic markers remained unchanged, we found increased expression of PSD-95 in S49P-Syracuse transgenic mice compared to wild-type mice. This effect is caused by the S49P mutation and is not a protein overexpression artifact, since in mice overexpressing wild-type neuroserpin no dysregulation of PSD-95 expression can be observed. Since a decrease in synaptic markers is seen when synapses are lost, this result rather argues for absence of synaptic toxicity in FENIB mice at this stage. This is in line with the results described before for hippocampal neurons where we did not detect signs of synaptotoxicity. Moreover, since in other neurodegenerative diseases loss of synapses is accompanied by astrogliosis and neuroinflammation, the absence of synaptotoxicity in S49P-Syracuse transgenic mice is in line with the very mild and transient inflammatory response described for this FENIB mouse model, by far weaker than in Alzheimer’s disease, tauopathy and Creutzfeldt-Jakob disease mouse models^15^. Still, a rise in PSD-95 was quite unexpected. Similarly, we previously described in adult neuroserpin-deficient mice increased PSD-95 expression, together with a reduction in density of synapses, impairment in long-term potentiation, and alterations in hippocampal-dependent behavioral tasks^3^. We speculate that in both FENIB and neuroserpin-deficient mice a rise in PSD-95 expression could represent a compensation for synaptic damage. Alternatively, in view of the heterogeneity of synapses regarding the expression of synaptic markers, we can hypothesize that at this disease stage, rather immature, PSD-95-negative synapses are lost. Further investigations are needed to elucidate the synaptic phenotype in the FENIB mouse model.

In conclusion, we show that a fraction of G392E-mutant neuroserpin is secreted via the ER-to-Golgi pathway, but this secreted mutant protein is per se not toxic to neuronal cells and in particular not synaptotoxic. Still, we cannot exclude that under more physiological conditions mutant neuroserpin-induced synaptotoxicity could play a role in the pathogenesis of FENIB.

## Supplementary Information

Supplementary Information.

## Data Availability

The datasets generated and analyzed during the current study are available from the corresponding author.

## Abbreviations

α1-AT: α1-Antitrypsin
AU: Arbitrary units
BFA: Brefeldin A
DIV: Days in vitro
EndoH: Endoglycosidase H
ER: Endoplasmic reticulum
ERAD: ER-associated protein degradation
FENIB: Familial encephalopathy with neuroserpin inclusion bodies
LDH: Lactate dehydrogenase
MAPS: Misfolded-associated protein secretion
PBS: Phosphate buffered saline
PNGaseF: *N*-Glycosidase F
PSD-95: Postsynaptic density protein 95
RCL: Reactive center loop
Syr-Tg: Transgenic mice overexpressing human neuroserpin carrying the S49P-Syracuse mutation
tPA: Tissue plasminogen activator
UPR: Unfolded protein response
USP19: Ubiquitin specific peptidase 19
WT-Tg: Transgenic mice overexpressing wild-type human neuroserpin
TPS: Total protein stain
