# Supplementary material for: G392E neuroserpin causing the dementia FENIB is secreted from cells but is not synaptotoxic
Source: Sci Rep. 2021 Apr 22;11:8766. doi: 10.1038/s41598-021-88090-1 (PMC8062559; doi:10.1038/s41598-021-88090-1)
Supplement: Supplementary file 1 — Supplementary Information. [file 41598_2021_88090_MOESM1_ESM.pdf]

Supplementary material

G392E neuroserpin causing the dementia FENIB is secreted from cells but is not synaptotoxic

Thies Ingwersen[#,1,2], Christian Linnenberg[#,1], Emanuela D'Acunto [3], Shabnam Temori [1], Irene Paolucci [1], David Wasilewski [1], Behnam Mohammadi [1], Johannes Kirchmair [4,5], Robert C. Glen [4,6], Elena Miranda [3,7], Markus Glatzel [1], Giovanna Galliciotti [1,*]

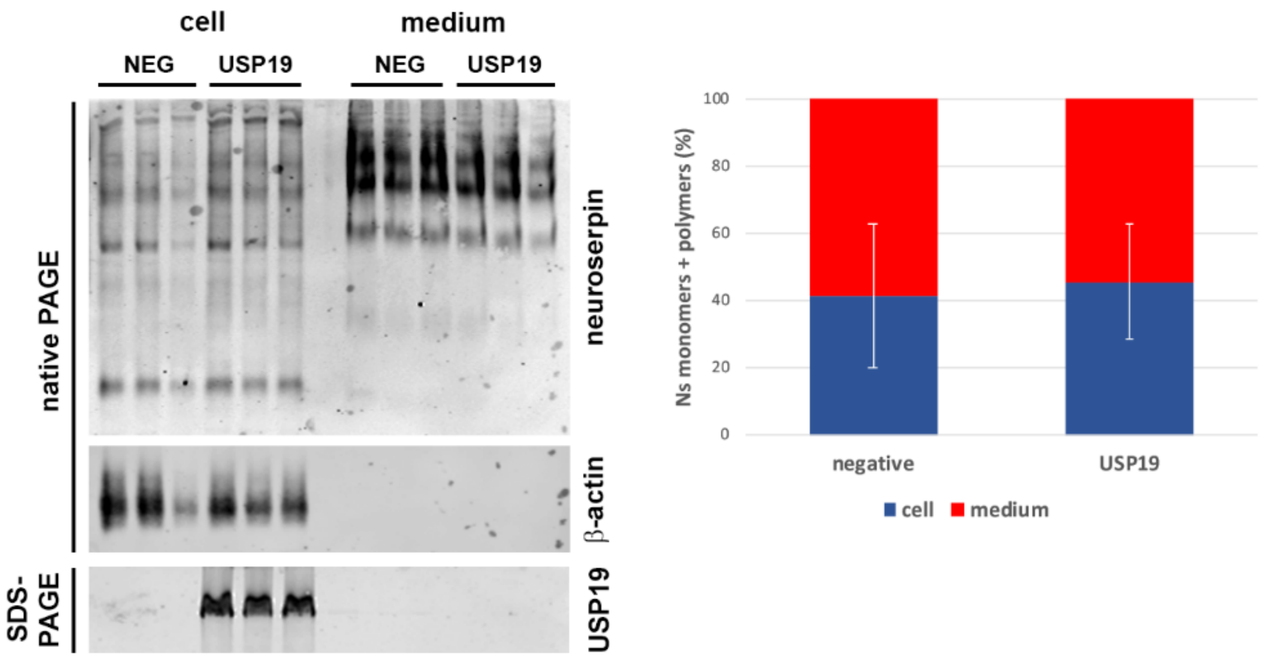

Supplementary Figure S1: Overexpression of USP19 does not increase G392E neuroserpin secretion.

HEK-293 cells overexpressing G392E-mutant neuroserpin were transiently transfected with a construct encoding mCitrine-USP19. After overnight incubation, cell extracts (cell) and culture media (medium) were collected and analyzed by non-denaturing PAGE followed by western blot using an anti-neuroserpin antibody. Beta-actin was used as loading control. In parallel, the same samples were subjected to SDS-PAGE and western blot analysis with anti-GFP antibody to assess the efficiency of mCitrine-USP19 transfection (bottom panel, USP19). The bar graph shows densitometric quantification of neuroserpin signal. Intensity revealed unchanged distribution of G392E-mutant neuroserpin in cells overexpressing mCitrine-USP19, implying that MAPS is not involved in secretion of mutant neuroserpin. Three independent experiments with three technical replicates each were performed and a representative one is shown. Values are mean +/- SD; n=3; p=0,6675.

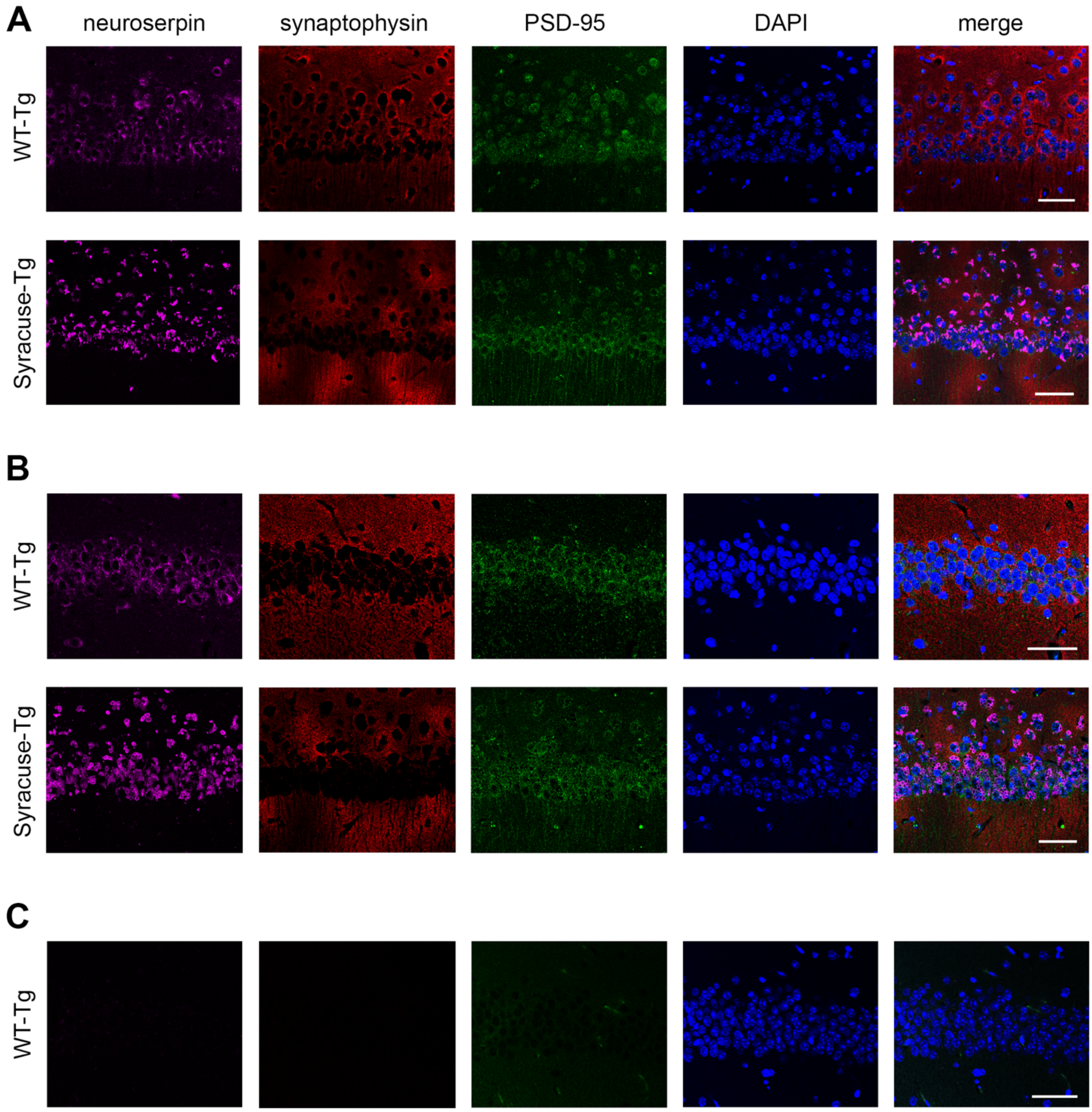

Supplementary Figure S2: Immunohistochemical stainings of neuroserpin, the pre-synaptic marker synaptophysin and the post-synaptic marker PSD-95 in the CA1 region of the hippocampus of transgenic mice overexpressing wild-type or S49P-Syracuse mutant neuroserpin.

Mice aged 45 (A) and 80 weeks (B) were analyzed. In C, as a negative control, sections from an 80 weeks old mouse were stained with the secondary antibody only. Scale bar, 50 μm.

uncropped membranes shown in figure 1

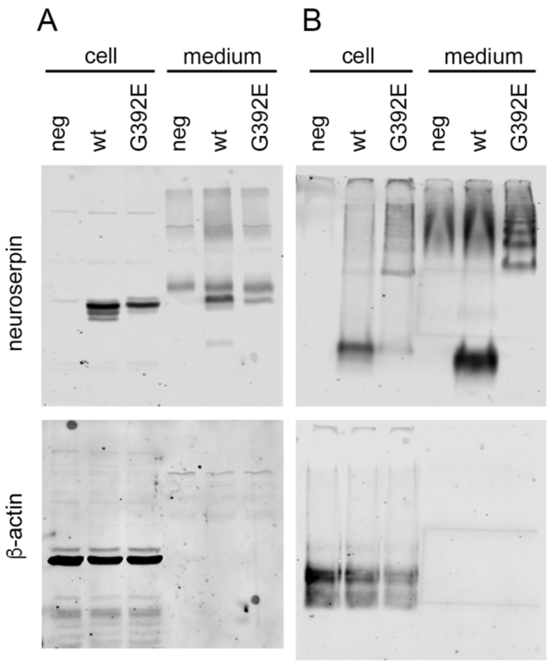

uncropped membranes shown in figure 2

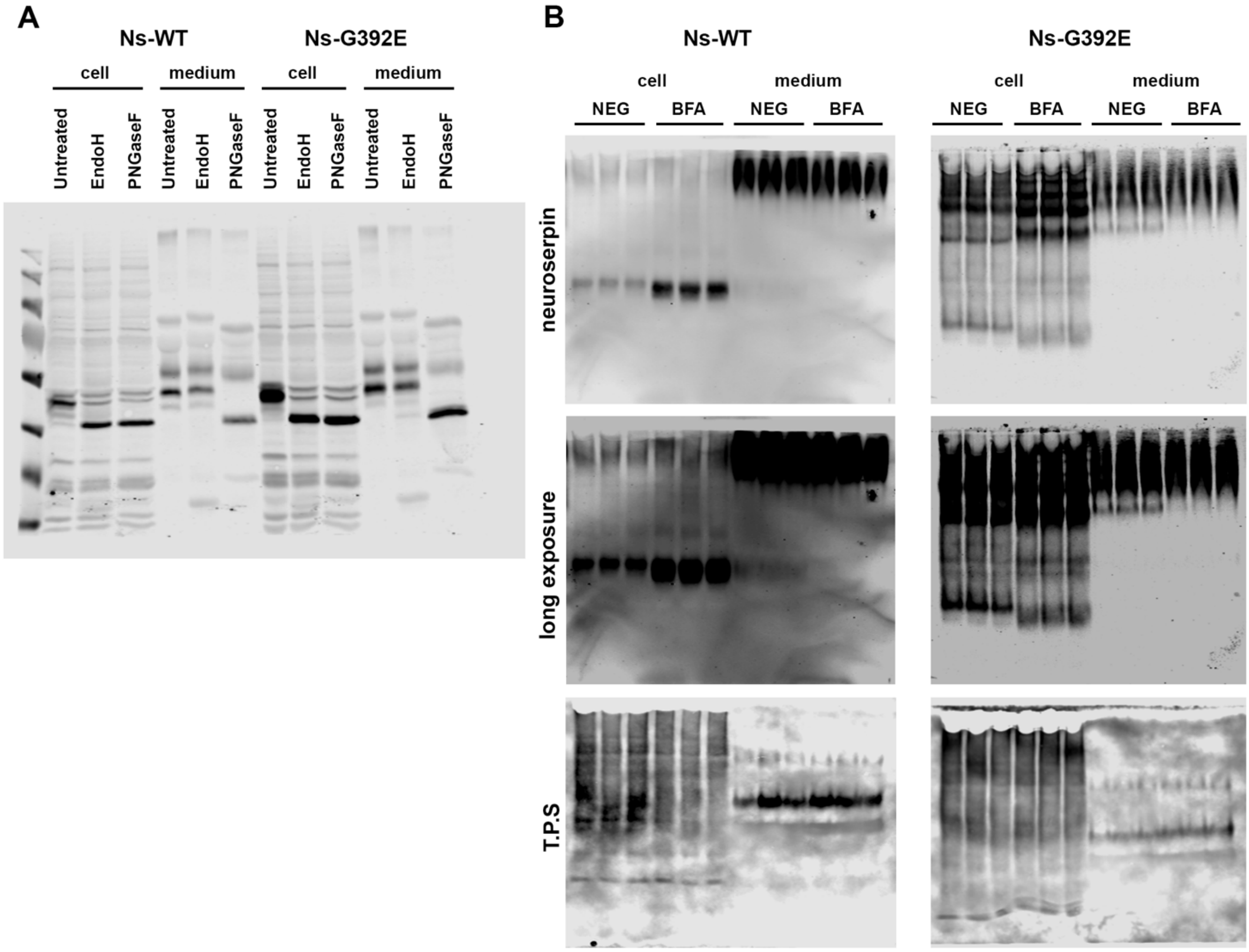

uncropped membranes shown in figure 3

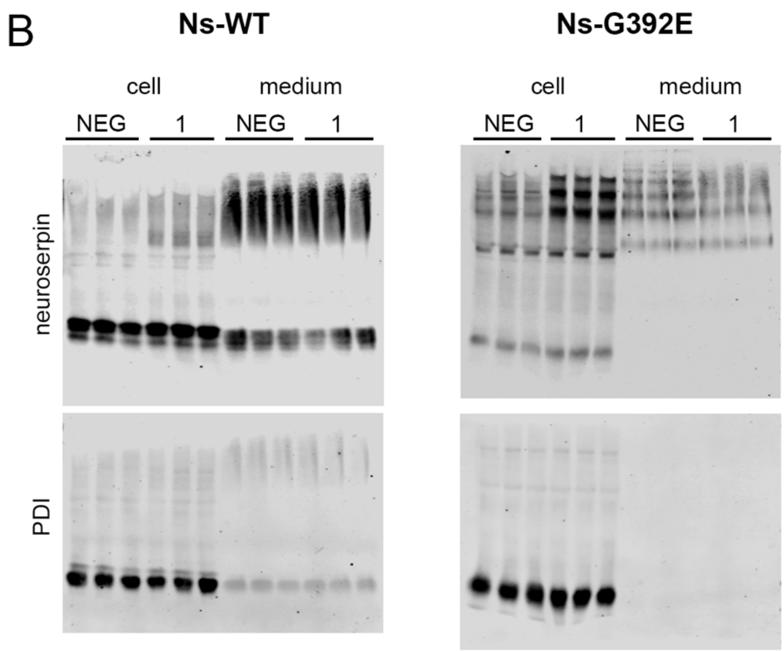

uncropped membranes shown in figure 5

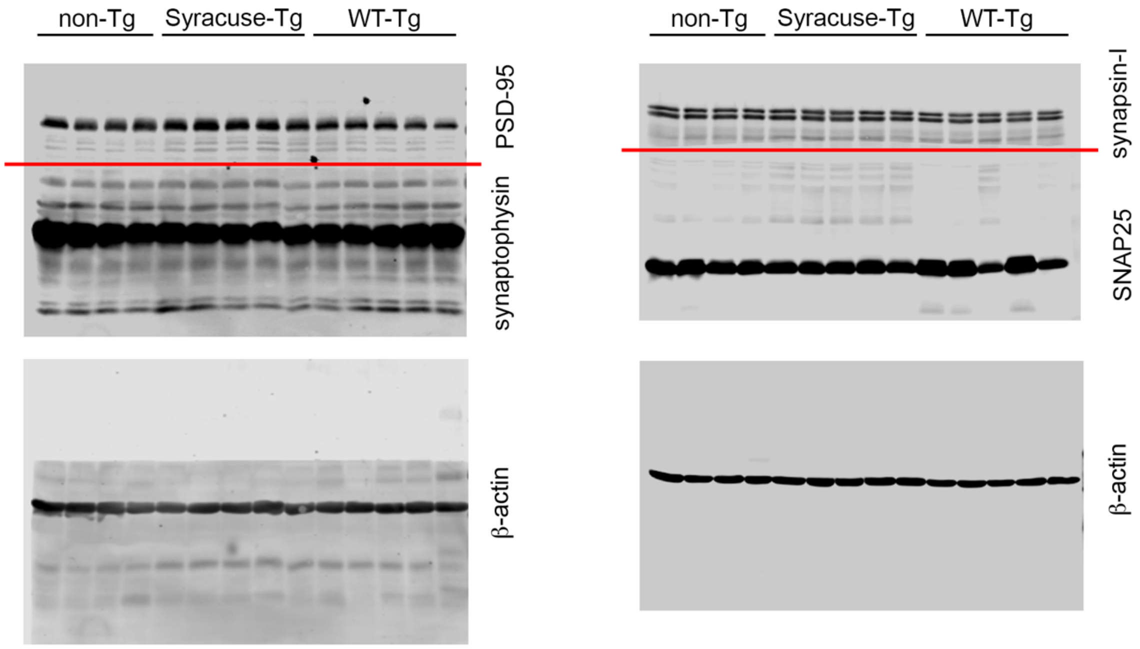

Supplementary Figure S3: Full-length blots.

Shown are the full-length blots depicted in Figure 1a, 1b, 2a, 2b, 3b and 5 before cropping. After blotting, the membranes of Figure 5 were cut in two pieces (s. red line), the upper part was probed with antibodies against PSD-95 or synapsin-I, the lower part with antibodies against synaptophysin, SNAP25 and beta-actin.

LDH assay									
	1	2	3	4	5	6	average	SD	p-value (One-way ANOVA)
negative	0,2943	0,2930	0,2810	0,2777	0,2710	0,2877	0,2841	0,0092	0,4472
wild-type	0,2890	0,2800	0,2770	0,2823	0,2780	0,2680	0,2791	0,0069	
G392E	0,2880	0,2823	0,2803	0,2790	0,2860	0,2805	0,2827	0,0036	

Cleaved caspase 3									
	1	2	3	4	5	6	average	SD	p-value (One-way ANOVA)
negative	0,2700	0,2200	0,4300	0,3206	0,1320	0,5089	0,3136	0,1381	0,3146
wild-type	0,3540	0,3460	0,4640	0,5739	0,4348	0,1214	0,3824	0,1526	
G392E	0,2040	0,4280	0,9300	0,4054	0,4848	0,4244	0,4794	0,2409	

Synaptic puncta - recombinant neuroserpin (20nM)									
Puncta density / Area	1	2	3	4	5	6	average	SD	p-value (One-way ANOVA)
negative	1,4203	0,6624	0,7040	1,0913	0,8646	1,2575	1,0000	0,3070	0,3030
wild-type	2,0891	0,7856	1,2407	1,1440	0,7064		1,1932	0,5501	
G392E	1,6770	0,9739	1,7225	1,7396	0,8799	1,3373	1,3884	0,3876	
Puncta Area	1	2	3	4	5	6	average	SD	p-value (One-way ANOVA)
negative	1,2013	0,8371	1,0482	0,8632	1,1570	0,8932	1,0000	0,1576	0,5177
wild-type	1,4243	0,9531	1,0850	0,9112	1,0950		1,0937	0,2015	
G392E	1,4675	1,0011	0,9531	1,0131	1,1330	1,1344	1,1170	0,1868	

Synaptic puncta - recombinant neuroserpin (100nM)									
Puncta density / Area	1	2	3	4			average	SD	p-value (One-way ANOVA)
negative	1,0674	0,6504	1,1028	1,1794			1,0000	0,2377	0,9810
wild-type	0,8272	0,7211	1,3107	1,1398			0,9997	0,2731	
G392E	0,7902	0,5988	1,1863	1,2889			0,9661	0,3258	
Puncta Area	1	2	3	4			average	SD	p-value (One-way ANOVA)
negative	1,1037	0,8398	1,0062	1,0502			1,0000	0,1140	0,4255
wild-type	1,0857	1,0857	1,0257	1,4636			1,1652	0,2009	
G392E	1,0377	0,8638	1,2922	1,1757			1,0924	0,1845	

Synaptic puncta - HEK-293 co-culture									
Puncta density / Area	1	2	3	4	5	6	average	SD	p-value (One-way ANOVA)
negative	2,0028	1,2711	1,0543	0,4996	0,4332	0,7389	1,0000	0,5868	0,4790
wild-type	2,3429	2,0099	1,0927	0,7295	1,1615	1,1601	1,4161	0,6193	
G392E	1,8757	1,8401	1,0494	1,1236	0,6789	0,7176	1,2142	0,5287	
Puncta Area	1	2	3	4	5	6	average	SD	p-value (One-way ANOVA)
negative	1,7480	1,1216	0,8160	0,8012	0,6676	0,8457	1,0000	0,3955	0,8181
wild-type	1,4599	1,3110	1,0311	0,8838	0,9792	1,0682	1,1222	0,2182	
G392E	1,0682	1,6913	0,8833	1,3575	0,6053	0,9235	1,0882	0,3844	

Supplementary Table S1: Lack of cell and synaptic toxicity of G392E-mutant neuroserpin in primary hippocampal neurons.

Data obtained from treatments of primary hippocampal neurons with either human recombinant neuroserpin or by co-culture with HEK-293 cells overexpressing neuroserpin. Neuronal toxicity was analyzed by LDH activity in the culture medium and caspase 3 activation. For analysis of synaptic toxicity (density and area of synaptic puncta) value for the negative control was set to 1.
